# Mediterranean Diet Can Reduce Fat Accumulation and Obesity Progression Through Action of Plant Bioactive Molecules

**DOI:** 10.3390/ijms27094134

**Published:** 2026-05-05

**Authors:** Paola Sportiello, Miriam Piccioni, Vito Flavio Licciulli, Giuseppe Cananzi, Stefania Crispi, Domenico Catalano

**Affiliations:** 1 Institute of Biosciences and BioResources—CNR SS of Naples, Via P. Castellino 111, 80131 Naples, Italy; paola.sportiello@unina.it (P.S.); miriampiccioni@cnr.it (M.P.); 2 Department of Biology, University of Naples Federico II, Via Cupa Nuova Cinthia, 80126 Naples, Italy; 3 Institute for Biomedical Technologies—Consiglio Nazionale delle Ricerche, Division of Bari, Via G. Amendola 122/D, 70126 Bari, Italy; flavio.licciulli@cnr.it (V.F.L.); giuseppe.cananzi@cnr.it (G.C.); domenico.catalano@cnr.it (D.C.)

**Keywords:** obesity, Mediterranean diet, plant bioactive molecules, plant miRNAs

## Abstract

Obesity is a multifactorial disease associated with a chronic imbalance between energy intake and energy consumption, as well as the ingestion of high-fat foods. It is widely reported that the Mediterranean Diet (MD), a dietary regimen rich in vegetables, fruits, fiber and complex polyunsaturated lipids, can positively act on obesity onset. These aliments contain bioactive molecules that exert beneficial effects on two traits often associated with obesity: lipid accumulation and imbalance in oxidative homeostasis. Additionally, they can act on metabolic pathways linked to obesity through the cross-kingdom activity of plant miRNAs. In this review, we provide an overview of the studies describing the anti-obesogenic effect of plant-based foods typical of the Mediterranean Diet. We describe the results of recent studies that link the effect of lipid reduction with the ingestion of bioactive molecules or plant miRNAs typical of MD foods. We also report how advances in bioinformatic analyses have elucidated the role of plant-derived miRNAs in metabolic homeostasis, revealing how the cross-kingdom interaction results in the anti-obesogenic action of the MD. These findings shed light on the molecular mechanisms through which the MD dietary pattern exerts its metabolic effects, suggesting new perspectives on MD nutrition-based strategies as novel therapeutic approaches for obesity.

## 1. Introduction

Obesity is widely recognized as one of the major public health challenges of the 21st century. The World Health Organization defines obesity as an excessive or abnormal accumulation of body fat that poses a significant risk to health. It is strongly linked to a higher likelihood of developing chronic conditions such as type 2 diabetes, cardiovascular diseases, non-alcoholic fatty liver disease, and several types of cancer [1]. The global burden of obesity has grown dramatically in recent years, and in 2022, more than one billion people worldwide were estimated to be living with obesity, corresponding to roughly 16% of adults and 8% of children and adolescents [2,3]. Nowadays, obesity affects populations across virtually all regions, age groups, and socioeconomic strata [4,5], and it is expected to increase in the next 10 years [6]. Obesity is a multifactorial disorder arising primarily from a sustained imbalance between energy intake and expenditure. Although unhealthy dietary patterns, particularly those rich in ultra-processed foods and sugar-sweetened beverages, together with low physical activity, are major contributors, they do not fully explain inter-individual variability in susceptibility to weight gain and metabolic complications [7,8,9].

Dietary patterns rich in plant-based foods and with balanced caloric intake, such as the Mediterranean Diet (MD), have been shown to prevent or reduce adipose tissue accumulation [7,8]. These diets act directly through bioactive compounds by modulating biological processes and overall health [9,10] or indirectly through the action of plant microRNAs (miRNAs).

The Mediterranean Diet, a term introduced by Ancel Keys in 1960 [11], is one of the most extensively studied dietary patterns worldwide. Its origin lies in the traditional habits of populations from Mediterranean regions, where dietary practices developed alongside local lifestyles and social customs. This cultural and geographical connection forms the basis for the coherence between the diet’s composition and the health benefits observed. Consequently, the MD has been recognized by UNESCO as an “Intangible Cultural Heritage”, representing a set of agricultural and dietary practices deeply rooted in the culture and territory, developed in harmony with the surrounding environment typical of the Mediterranean basin [12].

A high intake of fruits, vegetables, whole grains, legumes, extra virgin olive oil, and aromatic herbs has been associated with reduced abdominal fat accumulation [13,14]. Scientific evidence indicates that such plant-derived foods exert beneficial effects on liver metabolism, lowering lipid accumulation, oxidative stress, and inflammation, while also positively modulating gut microbiota composition [15,16,17].

Dietary components, such as vitamins, polyphenols, and fatty acids, and specific dietary patterns can regulate metabolism by modulating endogenous miRNA expression [18,19]. Additionally, exogenous dietary miRNAs may be absorbed during digestion and directly affect host gene expression [20,21]. Plant-derived miRNAs can exert cross-kingdom effects, contributing to host metabolic regulation and the homeostasis of diverse biological processes [22]. These miRNAs can modulate gene expression in host cells and regulate cell survival, proliferation, and differentiation, as well as metabolism, immune responses, and some specific pathways linked to obesity, including adipocyte differentiation, adipokine production, and glucose and lipid metabolism [23].

Experimental evidence suggests that adherence to the Mediterranean Diet can modulate the expression of specific miRNAs, positioning them as potential mediators of the diet’s anti-inflammatory and antioxidant effects. While the extent and physiological relevance of cross-kingdom transfer remain under investigation, these findings open new perspectives for nutrition-based interventions that can be viewed as novel anti-obesogenic therapeutic approaches [24].

This review aims to synthesize current knowledge on the molecular mechanisms involved in obesity development, with particular emphasis on the influence of the MD dietary pattern. It focuses on plant-derived foods typical of the Mediterranean Diet and the bioactive compounds that contribute to their metabolic effects. Within this context, the emerging role of microRNA-mediated regulation, encompassing both endogenous and diet-associated miRNAs, is discussed as a potential link between nutrition and key processes such as inflammation, oxidative stress, and metabolic regulation.

## 2. Environmental and Biological Factors of Obesity

Metabolic activity of gut microbes is crucial for maintaining host health and mediating energy homeostasis [25]. Also, the preponderance of specific bacterial species such as Firmicutes (F) and Bacteroides (B) is pivotal in metabolic diseases, and an imbalance between these two phyla, with an increased F/B ratio, has been associated with an increase in body weight and obesity [26].

MD food components, such as microbiota-accessible carbohydrates (MACs), polyphenols, and polyunsaturated fatty acids (PUFAs), are able to modulate microbiota composition and metabolism by increasing bacterial strains producing short-chain fatty acids (SCFAs) [27].

SCFAs, a group of saturated fatty acids containing up to six carbons, are produced by gut microbes during fermentation and can modulate host biological responses both at the cellular and molecular levels [28]. SCFAs are known to improve gut physiology and modulate host glucose and lipid metabolism [29], but they also affect immune function by inhibiting histone deacetylases (HDACs) [30]. The effects of SCFAs on metabolism are exerted through the activation of G-protein-coupled receptors (GPCRs) GPR41, GPR43, and GPR109, which turn on AMP-activated protein kinase (AMPK), a protein that modulates lipid metabolism [31]. SCFA activation of GPR41 and GPR43 affects glucose metabolism and fat accumulation [32,33], while activated GPR109 is involved in lipolysis and a plasma decrease in free fatty acids [34].

Changes in gut microbiota composition can also be linked to metabolic diseases through the release into the bloodstream of the bacterial endotoxin lipopolysaccharide (LPS) [35]. In fact, in these cases, the alteration in gut permeability allows the transfer of LPS from the intestine to blood circulation [36]. The prolonged presence of circulating LPS in the body determines the onset of metabolic endotoxemia (ME). In this condition, LPS in the adipose tissue enables the entry of macrophages and the initiation of the inflammatory cascade [37]. The inflammatory cascade starts with the binding of LPS to toll-like receptor family 4 (TLR4) and the subsequent activation of nuclear factor kappa B (NFκB), a transcription factor that regulates genes involved in many aspects of the inflammatory response [38,39]. The elevated presence of TLR4 in adipose tissue strengthens its role in the regulation of lipid homeostasis.

ME, by eliciting a chronic low-grade pro-inflammatory and pro-oxidative stress status, can account for the link between obesity, inflammation and type 2 diabetes [40]. ME is often associated with a high-fat diet, characteristic of the Western diet [41], and several studies have reported that a high-fat diet induces gut dysbiosis associated with an increase in LPS levels, which can be lowered by the ingestion of foods rich in PUFAs [42,43].

There is growing evidence that obesity is associated with increased oxidative stress and that certain dietary components of the MD are able to reduce inflammation [44]. In this scenario, nuclear factor erythroid 2-related factor 2 (Nrf2), the master regulator of redox homeostasis, has a central role. Under obesogenic conditions, Nrf2 activation may initially serve as a compensatory mechanism to limit oxidative damage and maintain cellular function. However, when its activation becomes prolonged or dysregulated, it can disrupt redox balance and further contribute to metabolic dysfunction and obesity progression [45]. The imbalance in redox signaling provides a mechanistic link connecting metabolic changes, chronic low-grade inflammation, and alterations in gene expression. Also, the dietary modulation of Nrf2 has been associated with the intake of MACs, polyphenols, and PUFAs, which are able to influence the intestinal ecosystem and promote beneficial effects on host health [28]. Consequently, environmental and biological factors, through modulation of the gut microbiota, represent a key mechanistic pathway by which obesogens can amplify or mitigate the host’s genetic susceptibility to fat accumulation, contributing to the observed inter-individual variability in metabolic phenotypes and disease risk.

## 3. Physiology of Obesity

### 3.1. Lipid Metabolism and Obesity

**Figure 1:**
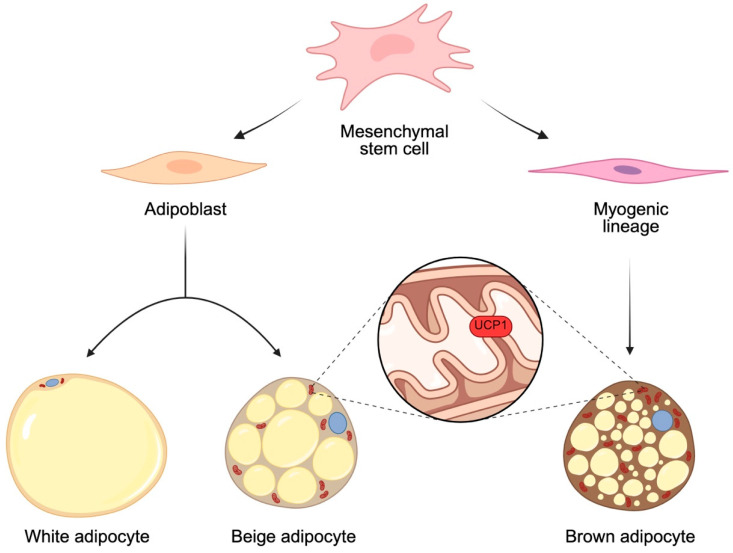
Adipocyte differentiation. The process of adipocyte differentiation begins with mesenchymal stem cells that serve as embryonic precursors for adipoblasts and cells of the myogenic lineage. White and beige adipocytes derive from the same progenitor and reside in the same depots but have different characteristics. White adipocytes have a single large lipid droplet (yellow) which confines the nucleus (blue) and few mitochondria (red) at the edges of the plasma membrane and have fat storage functions. Beige adipocytes contain many lipid droplets and abundant mitochondria. Brown adipocytes differentiate from progenitors of the myogenic lineage and have a very high number of lipid droplets and mitochondria. Beige and brown adipocytes both contain UCP1 in their mitochondria, also known as thermogenin, a protein which makes these adipocytes involved in the thermogenesis process. Dashed lines represent an enlargement of mitochondrial cristae with a focus on UCP1. Created in BioRender by Piccioni, M. (2026), BioRender.com/vesb9cn.

Adipose tissue (AT) is a dynamic and large organ that performs a complex endocrine function, secreting adipokines and cytokines that influence insulin sensitivity, inflammation and lipid homeostasis. Adipocytes, also known as fat cells, are the main functional cell type of adipose tissue. In mammals, there are various types of adipocytes, such as white, brown and beige (Figure 1).

White adipocytes (WATs) store lipids such as triglycerides, while brown adipocytes (BATs), through the presence of uncoupling protein-1 (UCP1), are involved in thermogenesis regulation. Beige adipocytes, known as “brown-in-white adipocytes”, exhibit multilocular lipid droplets and the expression of UCP1, like brown adipocytes, but reside in WAT depots [46]. AT is no longer considered a simple fat depot but a metabolically active tissue that regulates energy balance, and it is widely recognized as a key organ in energy homeostasis and obesity onset [47]. A state of positive energy balance occurs when energy intake exceeds energy expenditure, and this determines an increase in body mass, usually body fat [48].

**Figure 2:**
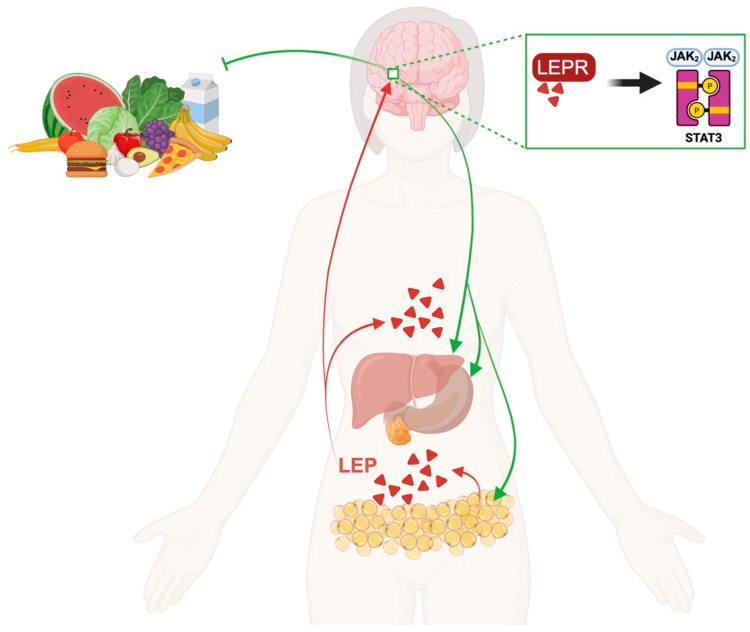
Leptin negative feedback. LEP negative feedback begins (red arrows) with its secretion into adipose tissue. LEP crosses the BBB via receptor-mediated transport and binds to its receptor in the hypothalamus. This binding activates the JAK2/STAT3 pathway (green arrows), which in turn modulates satiety, in addition by influencing peripheral organs. Created in BioRender by Piccioni, M. (2026), BioRender.com/pvzgbua.

Energy homeostasis is achieved by a very complex system in which peripheral signals inform the brain of available energy stores, and, in response, corrective adjustments to food intake are made by the brain [49]. A classic regulatory mechanism of this “adiposity negative feedback” is represented by the adipocyte hormone leptin, also called the “satiety hormone” (Figure 2).

Leptin (LEP) is secreted from adipose tissue, and through receptor-mediated transport, it crosses the blood–brain barrier, reaching the central nervous system. By binding to receptor leptin receptor 8 (LEPR), leptin then activates the Janus Kinase 2/Signal Transducer and Activator of Transcription 3 (JAK2/STAT3) pathway, which regulates the production of different anorexigenic neuropeptides, such as Cocaine- and Amphetamine-Regulated Transcript (CART) and Pro-opiomelanocortin (POMC) [50,51]. The leptin-induced signal enhances the response to satiety by inhibiting food intake and increasing energy expenditure, thus affecting obesity.

At the biochemical level, lipid accumulation depends on de novo lipogenesis, lipid mobilization from adipose deposits, and lipid degradation, which occurs through β-oxidation. These processes are the key factors in the metabolic physiopathology of obesity [52].

In healthy individuals, lipids play a fundamental role not only as an energy reserve but also as structural components of cell membranes and signaling molecules. The synthesis of lipids occurs primarily in the cytosol of hepatocytes and adipocytes, starting from fatty acid precursors generating lipid droplets (LDs) [53]. In lipolysis, the triglycerides accumulated in LDs are hydrolyzed into fatty acids and glycerol, allowing their transport and use for energy.

**Figure 3:**
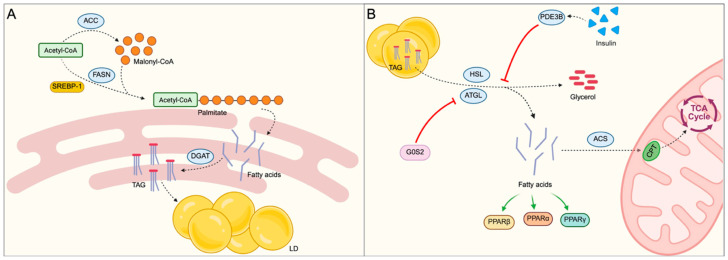
Lipid metabolism. (**A**) Lipid synthesis begins in the cytosol, where acetyl-CoA is utilized by fatty acid synthase (FASN) to produce palmitate. This process is transcriptionally regulated by SREBP1. Palmitate is subsequently elongated and desaturated in the endoplasmic reticulum to generate a variety of fatty acids. Fatty acids are activated and incorporated into triglycerides (TAGs) by diacylglycerol acyltransferase (DGAT), and these are then stored in lipid droplets (LDs). (**B**) During lipolysis, triglycerides stored in LDs are hydrolyzed into glycerol and fatty acids through the action of adipose triglyceride lipase (ATGL) and hormone-sensitive lipase (HSL). In β-oxidation, fatty acids are activated by acyl-CoA synthetases (ACSs) and transported into mitochondria via the carnitine shuttle (CPT), where they fuel the tricarboxylic acid (TCA) cycle. Created in BioRender by Piccioni, M. (2026), BioRender.com/m2pecd9, BioRender.com/xjb0b9p.

The complexity of lipid metabolism (Figure 3) underlies how alterations affecting lipid synthesis, accumulation, and oxidation may contribute to the development and progression of obesity, highlighting the crucial role of lipid metabolism in the onset of associated metabolic complications.

### 3.2. Genetics of Obesity

Genetic predisposition plays a crucial role in susceptibility to obesity, although genetic variants account for only a limited fraction of the phenotypic variability in obesity.

Genome-wide association studies (GWASs) and integrated “omics” analyses have mapped hundreds of genetic loci associated with obesity, highlighting its polygenic and complex nature. These studies, through the identification of genetic subtypes with distinct risk profiles, indicate that genetic variants not only influence the obesity trait itself but also metabolic comorbidities [54].

Genetic variants of obesity include both rare monogenic and complex polygenic forms. Monogenic forms of obesity are generally caused by mutations in genes involved in the central regulation of appetite and energy balance, such as those encoding *LEP*, *LEPR*, melanocortin 4 (*MC4R*), and *POMC*. Mutations in the *LEP* and *LEPR* genes result in early-onset severe obesity, characterized by hyperphagia and rapid weight gain [55]. The *MC4R* gene represents the most common monogenic cause of childhood obesity [56]. The crucial role of melanocortin and therefore of its receptor in regulating appetite and energy expenditure results from the activation of POMC neurons through LEPR, thus confirming the importance of the leptin–melanocortin axis. *POMC* encodes a protein that influences the leptin–melanocortin system, resulting in appetite suppression, and its depletion leads to hyperphagia and decreased metabolic activity, resulting in severe obesity [57].

Although monogenic forms of obesity exist, most cases are polygenic and are determined by small variations at many loci, with cumulative contributions from environmental factors. More recently, GWASs have significantly expanded the number of known variants, and some studies, including multi-trait and multi-ethnic analyses, have attempted to decipher how combinations of variants increase the risk of obesity and related metabolic disorders [54]. GWASs have identified hundreds of loci associated with the body mass index (BMI) and body fat distribution. Among these, a single-nucleotide polymorphism (SNP) in the *FTO* gene represents one of the strongest genetic associations with BMI [58]. Variants in *FTO* are often linked to childhood obesity and severe adult obesity, influencing adiposity through modulation of food intake and eating behavior rather than energy expenditure [59].

A recent emerging development in the genetics of obesity is the use of the Polygenic Risk Score (PRS) to predict an individual’s risk of hereditary predisposition to developing a high BMI and obesity. The PRS, by analyzing thousands of SNPs, can potentially guide personalized strategies based on genetic predisposition [60].

### 3.3. Epigenetic Regulation of Obesity

Epigenetic modifications represent another factor that, together with nutritional, genetic, and environmental factors, can regulate the metabolic pathways involved in obesity [61]. These mechanisms include DNA methylation, post-translational modifications of histones, and regulation mediated by non-coding RNAs, particularly miRNAs.

Recent evidence indicates that epigenetic alterations contribute to adipose tissue dysfunction, insulin resistance, and chronic low-grade inflammation typical of obesity. The methylation profile of DNA from young obese subjects indicates the presence of a high number of differentially methylated CpG sites related to obesity [62]. DNA methylation in specific genomic regions can modulate gene expression by directly hindering the binding of transcription factor complexes to DNA or by promoting histone modifications through the recruitment of proteins that recognize methylated CpG sites [63]. Furthermore, post-translational modifications of histones, including acetylation, methylation, and sumoylation, regulate gene activity by altering chromatin structure and thus its accessibility to transcriptional machinery.

Non-coding RNAs, particularly miRNAs, also play an important role in epigenetic regulation, contributing to the control of gene expression at the post-transcriptional level. miRNAs are small non-coding RNA molecules approximately 21–24 nucleotides long, highly conserved across species, which modulate the expression of target genes primarily through binding to the 3′UTR region of specific target mRNA sequences, resulting in translation repression or transcript degradation [64].

In the context of obesity, several miRNAs are dysregulated in white adipose tissue, the liver, and systemic circulation, contributing to the modulation of adipogenesis, lipogenesis, and lipolysis, as reported for miR-143, miR-103, and miR-107. miR-143 has been reported to be involved in adipocyte differentiation, suggesting its use as a potential therapeutic target for obesity and metabolic diseases [65]. Conversely, miR-103 and miR-107 may modulate insulin signaling by interfering with proteins involved in insulin receptor trafficking, contributing to the development of insulin resistance [66]. Other miRNAs linked to obesity are miR-34a, involved in lipid metabolism, whose overexpression resulted in increased triglyceride accumulation [67], and miR-33, implicated in cholesterol export and the inhibition of the expression of proteins involved in fatty acid β-oxidation [68]. The coordinated alteration in these miRNAs contributes to the transition from metabolically healthy adipose tissue to a pro-inflammatory and insulin-resistant state.

Finally, miRNAs act not only as intracellular regulators but also as mediators of metabolic crosstalk, and an emerging aspect concerns the “endocrine” function of circulating miRNAs. miRNAs can be transported to distant organs embedded in adipose tissue-derived extracellular vesicles (EVs), modulating gene expression, thus playing an important role in metabolism regulation [69].

## 4. MD as a Dietary Source of Anti-Obesogenic Molecules

The Mediterranean Diet represents a high-impact nutritional model for obesity prevention, due to its high content of bioactive plant-based molecules [70]. In addition to well-known polyphenols, the MD provides a wide range of functional compounds, including carotenoids, flavonoids, dietary fiber, phytosterols and miRNAs, which are involved in the anti-obesogenic effects of this dietary pattern.

The MD is characterized by a high intake of plant foods, such as fruits, vegetables, whole grains, beans, nuts and seeds, and wine, and the use of olive oil as the principal source of fat. The combination of these healthy foods not only provides essential nutrients but also generates a range of bioactive compounds that interact with human metabolism, conferring multiple health benefits, including the prevention of chronic diseases [71].

### 4.1. Dietary Plant Compounds Targeting Obesity

**Figure 4:**
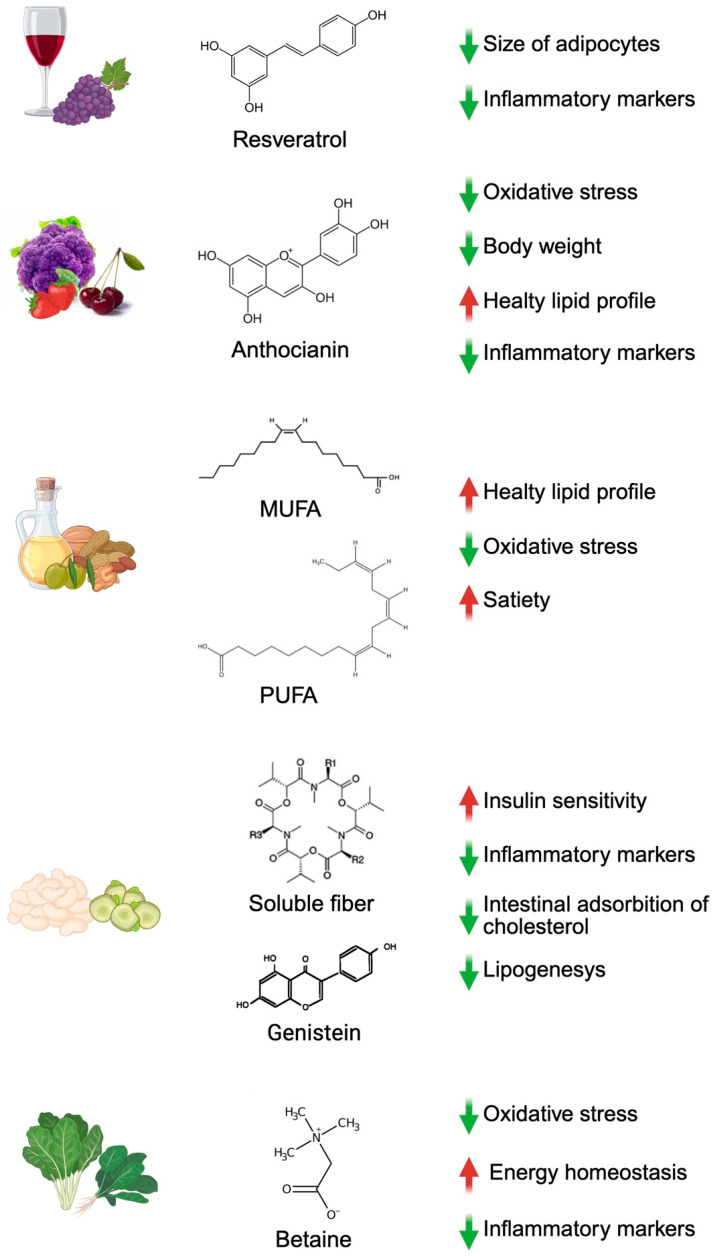
Main plant-based bioactive compounds in MD. Resveratrol is associated with a reduction in adipocyte size, inflammatory markers, and oxidative stress. Anthocyanins contribute to a reduction in oxidative stress, body weight, and inflammatory markers, with an improvement in lipid profile. MUFAs and PUFAs are associated with an improvement in lipid profile, a reduction in oxidative stress, and an increase in satiety. Soluble fiber and genistein promote insulin sensitivity and reduce inflammatory markers, intestinal cholesterol absorption, and lipogenesis. Betaine is associated with a reduction in oxidative stress and inflammatory markers, with a positive effect on energy homeostasis. Created in BioRender by Piccioni, M. (2026), BioRender.com/vt8cqws.

Plant-based foods typical of the Mediterranean Diet, owing to their rich bioactive composition and their ability to modulate gut microbiota, play a key role in the regulation of lipid metabolism, inflammation, and energy homeostasis, central mechanisms underlying obesity and its associated metabolic disorders. The MD, through the intake of fruits and vegetables, which are low-calorie foods, promotes prolonged satiety due to slower digestion times, reducing overall caloric intake [72] (Figure 4).

Vegetables and fruits in the MD are good sources of polyphenols, molecules associated with improvements in the lipid profile, including increased HDL (high-density lipoprotein) levels and reduced LDL (low-density lipoprotein) levels. Food-ingested polyphenols present in olive oil, fruits, vegetables, and red wine can reduce lipid peroxidation, regulate gene expression and miRNAs related to lipid metabolism, and exert anti-inflammatory effects [63,73].

Extra virgin olive oil (EVOO), the principal source of fat in the MD, extracted from olives, is rich in polyphenols, responsible for EVOO’s beneficial effects. Oleuropein, a polyphenolic compound enriched in EVOO, and its metabolite, hydroxytyrosol, have powerful antioxidant activity. Administration of oleuropein in high-cholesterol-fed rats reduces body weight, fat mass and serum lipid levels through the activation of AMPK, which reverses the typical effects of obesity [74]. In addition, hydroxytyrosol was able to reduce obesity in high-fat diet-fed mice by altering the microbiota composition, and, interestingly, its beneficial effects were transferable through fecal microbiota transplantation [75].

Another polyphenol present in red wine, resveratrol, has been the focus of several clinical studies on human obesity. Studies on obese men have shown that moderate doses of resveratrol resulted in a reduction in the size of abdominal subcutaneous adipocytes and further metabolic improvements, including a reduction in the serum levels of inflammatory markers such as Interleukin-6 (IL-6) and Tumor Necrosis Factor-alpha (TNF-α) [76]. Quercetin, a flavonoid present in various typical MD plant foods such as apples, berries, red onions, grapes, cherries, broccoli and citrus, has been reported to reduce dyslipidemia and insulin resistance in a rat obesity model. In addition, chronic administration reduces the production of TNF-α from visceral adipose tissue, a molecule involved in the characteristic chronic pro-inflammatory status of this rat model [77].

Anthocyanins, present in different fruits and vegetables such as berries and cruciferous vegetables, regulate the cell cycle and can activate DNA repair pathways, promoting metabolic resilience and protection against oxidative stress [78]. Interestingly, anthocyanins such as cyanidin, malvidin, and peonidin can modulate obesity by alleviating oxidative stress and inflammation. It has been reported that mice fed with a high-fat diet followed by cherry anthocyanin supplementation showed a reduction in body weight and an improvement in lipid profiles, along with a simultaneous reduction in the inflammatory state typical of obesity, with downregulation of *TNF-α*, *IL-6*, and NF*-κ*B [79].

EVOO is also rich in monounsaturated fatty acids (MUFAs), which, similarly to PUFAs, such as Omega-3 fatty acids from nuts and seeds, positively influence blood lipid profiles by increasing HDL, reducing LDL and triglycerides, and regulating key enzymes such as lipoprotein lipase and HMG-CoA reductase [80]. An in vitro study showed that Omega-3 fatty acids can modulate leukocyte chemotaxis, adhesion molecule expression, and eicosanoid synthesis, favoring redox homeostasis by reducing pro-inflammatory cytokines and reactive oxygen (ROS) and nitrogen species (RNS) [81]. In addition, PUFAs can bind and stimulate GPCRs, playing an important role in regulating glucose metabolism and thus leading to increased secretion of a glucagon-like hormone that can limit postprandial hyperglycemia and influence satiety in mice [82].

Carotenoids are liposoluble pigments, usually red, orange or yellow, which have beneficial effects on lipid metabolism and risk of obesity. A study showed that lycopene, the major carotenoid present in tomato, was able to increase the mRNA expression of thermogenic and mitochondrial genes in the WATs of obese mice fed with a high-fat and high-fructose diet. Therefore, lycopene may have the potential to stimulate WAT browning and accelerate lipolysis [83]. The metabolic benefits of this conversion from white to beige adipocytes primarily concern the central role of mitochondria in regulating energy balance, a key aspect of obesity treatment. A similar mechanism is played by sulforaphane, an organosulfur compound found in cruciferous vegetables such as broccoli, Brussels sprouts, and cabbage. It has been shown that sulforaphane in mature 3T3-L1 adipocytes allows for increased expression of UCP1, a protein specific to brown adipocytes, thus promoting the browning process [84].

Dietary soluble fiber from legumes, fruits and vegetables plays an important role in lipid metabolism and body weight regulation. This fiber also contributes to a healthier lipid profile, lowering LDL levels and decreasing intestinal absorption of cholesterol [85]. In addition, fermentation of fiber by gut microbiota produces SCFAs, such as acetate, propionate and butyrate, which regulate energy homeostasis, inhibit lipogenesis and improve insulin sensitivity [86].

Vegetables included in the MD provide bioactive compounds capable of modulating miRNAs involved in obesity-related metabolic pathways. Among these, betaine-rich plant foods, notably spinach, beetroot, and Swiss chard, have been reported to have health-promoting properties and regulate host lipid metabolism [87]. In this context, a study performed in mice reported that a diet supplemented with betaine reduced obesity by influencing the gut microbiota-derived miR-378a family, a regulatory node implicated in lipid metabolism, autophagy, and energy homeostasis [88]. The presence of these foods within the MD thus aligns with emerging evidence showing that dietary betaine can attenuate obesity-associated metabolic impairments through miRNA-dependent mechanisms, complementing the antioxidant and anti-inflammatory actions already attributed to MD vegetables. Furthermore, the isoflavone genistein, although abundant in soy, is also present in chickpeas (*Cicer arietinum*), a traditional MD legume. Experimental data demonstrate that genistein suppresses miR-222, thereby promoting lipid degradation and reducing adipose-derived pro-inflammatory cytokines TNF-α and IL-6 [89].

The integration of the wide spectrum of phytochemicals in Mediterranean Diet plant-based foods may contribute to the regulation of lipid metabolism, inflammation and energy balance. Through coordinated modulation of oxidative stress, gene expression, gut microbiota and metabolic signaling pathways, these dietary components play a fundamental role in the prevention and management of obesity and related metabolic disorders.

### 4.2. Dietary Plant miRNAs Targeting Obesity

miRNAs are non-coding RNAs, approximately 22 nucleotides in length, which are involved in post-transcriptional regulation of gene expression. Several studies suggest that the health-promoting properties of vegetables present in the MD could be mediated also through miRNA cross-kingdom activity. The cross-kingdom activity of plant miRNAs is linked to their structures and differences in their biogenesis.

**Figure 5:**
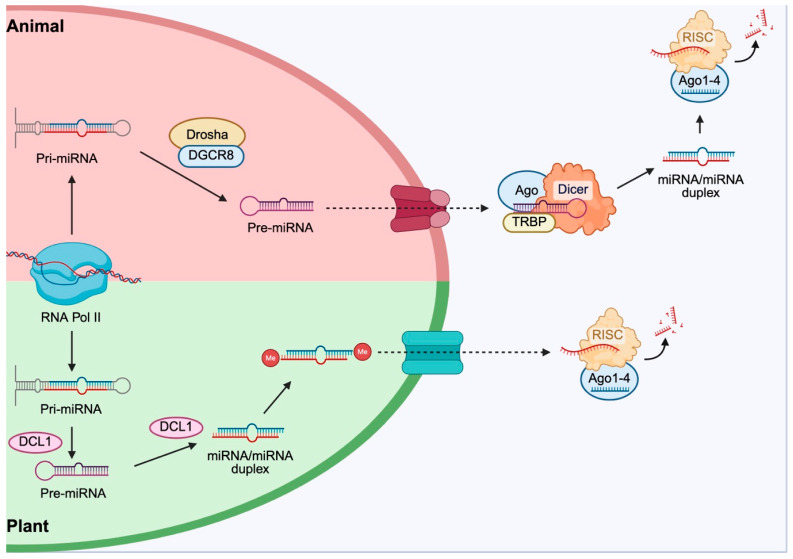
miRNA biogenesis in animals and plants. In animals, the biogenesis process occurs in the nucleus, where RNA polymerase II transcribes pri-miRNA. This is processed by the Drosha complex into pre-miRNA and exported to the cytoplasm, where it undergoes maturation by the Dicer complex. The generated duplex is incorporated into the RISC, mediating gene repression. In plants, pri-miRNA generated in the nucleus by RNA polymerase II is processed into pre-miRNA by the DCL1 enzyme and methylated before export to the cytoplasm. The duplex is incorporated into the RISC, responsible for gene silencing. Created in BioRender by Piccioni, M. (2026), BioRender.com/hzop5ne.

In animals, miRNAs are generally transcribed by RNA polymerase II, processed in the nucleus by the endonuclease Drosha, and subsequently matured in the cytoplasm by the enzyme Dicer before being incorporated into the RNA-induced silencing complex (RISC). In plants, miRNAs maturation occurs in the nucleus and is catalyzed by the enzyme DICER-LIKE 1 (DCL1), a homologue of the animal Dicer (Figure 5).

Plant miRNAs often show almost perfect complementarity with their targets, resulting in direct degradation of the target mRNA. In contrast, in mammals, complementarity is usually partial, and it leads primarily to translational repression [90].

miRNAs from edible plants can regulate gene expression in different organisms through dietary intake (cross-kingdom gene regulation), since they can resist the digestive process [91], acting as functional bioactive regulators of different biological processes [92].

The stability of plant miRNAs is mainly attributed to the methylation present at the 3′-end (2′-O), mediated by the enzyme HUA ENHANCER1 (HEN1), which prevents 3′ uridylation and 3′ truncation, and to their association with extracellular vesicles or protein complexes, which protect them from enzymatic degradation by improving their resistance to digestive processes and circulation [93]. In fact, several studies detected the presence of plant miRNAs in plasma and peripheral tissues in both humans and other mammals [94,95], indicating that their bioactivity is maintained even after cooking [96].

The first study reporting the cross-kingdom regulation of mammalian genes by plant miRNAs was performed in mice. The study indicated that a few hours after rice feeding, an elevated level of the rice miRNA miR-168a was found, along with a concomitant decrease in Low-Density Lipoprotein Receptor Adaptor Protein 1 (LDLRAP1), a gene involved in LDL removal. The decreased expression of LDLRAP1 led to an increase in plasma LDL cholesterol, indicating that food-derived miRNAs could have physiological relevance [20].

Beyond metabolic contexts, cross-kingdom gene regulation has been documented in different biological systems. For example, in vitro plant-derived miRNAs have been shown to target mammalian long non-coding RNAs such as MALAT1 and NEAT1, providing mechanistic evidence that dietary plant miRNAs can interact with human transcripts [97]. Although unrelated to obesity, this finding supports the biological feasibility of cross-kingdom interactions.

Other studies have reported that miRNAs present in edible plants can positively act against fat accumulation by regulating cholesterol homeostasis or fatty acid metabolism. A recent in vitro study showed that four miRNAs (miR-159a, miR-159b, miR-166a and miR-403) overexpressed in the crucifera *Brassica oleracea* var.italica (broccoli) are able to modulate cholesterol efflux, thus regulating cholesterol homeostasis [98].

Two further plant miRNAs, miR-8126-3p and miR-8126-5p, were reported to inhibit the expression of genes involved in lipid metabolism and triglyceride accumulation in a human hepatocyte model. These miRNAs led to a reduction in intracellular lipid accumulation, suggesting a potential protective role against obesity-associated metabolic diseases and dyslipidemia [99].

More recently, several miRNAs overexpressed in *Daucus carota* (carrots) were identified as regulators of genes involved in adipocyte fat accumulation, and their administration in vitro led to a reduction in intracellular triglyceride accumulation with a concomitant increase in glycerol release, indicating lipolysis [100]. Similarly, a pilot study on a miRNA pool extracted from *Moringa oleifera* reported that these miRNAs can modulate lipid metabolism and prevent metabolic dysregulation in pre-obese mouse models, highlighting a possible functional role of plant miRNAs in the control of lipid homeostasis [101].

Plant miRNAs can also act indirectly on lipid metabolism by modulating the gut microbiota. The gut microbiota is involved in lipid homeostasis, and a high-fat diet may lead to gut microbiota dysbiosis, impaired barrier function, and inflammation, thus promoting metabolic endotoxemia and insulin resistance. Food-ingested plant miRNAs, by modulating gut microbiota composition, act on the maintenance of lipid homeostasis. In this context, it has been demonstrated that dried nuts contain two conserved plant miRNAs, miR-156c and miR-159a, which display high complementarity toward the mammalian TNF receptor Tumor Necrosis Factor Receptor Superfamily Member 1A (Tnfrsf1a). These miRNAs, delivered through plant-derived vesicles, were shown to attenuate TNF-α-mediated inflammatory signaling in mice, reduce the expression of pro-inflammatory cytokines, and improve insulin sensitivity in both adipocyte models and diet-induced obese mice [102].

Indirect effects of plant miRNAs from different vegetal sources can also influence lipid metabolism indirectly by modulating the gut microbiota. These miRNAs positively affect the growth of beneficial bacterial strains while reducing that of dangerous strains [103].

All these findings suggest that plant-derived microRNAs are potential bioactive components of foods, confirming that the action of diet-ingested miRNAs on the host’s gene expression could revolutionize the current paradigm of nutrition.

However, there are several controversies and limitations to consider. For instance, the cross-kingdom mechanism of action of dietary plant miRNAs has not yet been fully elucidated, and several studies have reported that cooking can lead to the degradation of plant miRNAs [104,105]. In addition, another question concerns how plant miRNAs can resist the extreme environment of the gastrointestinal tract while maintaining their stability [106].

As reported above, the stability of plant-derived miRNAs seems to be related to the presence of 2′-O-methylation at the 3′-end [93], and a study performed in mice on endogenous miRNAs indicated that this modification increased resistance to exonuclease degradation and digestive stability [107]. However, a replication of the first study on the cross-kingdom activity of rice miR-168a [20] was not able to detect the uptake of any rice miRNAs in mice after feeding them with standard chow, a raw rice diet or a diet supplemented with 41% rice [108]. Similarly, another study affirming the presence of circulating plant miRNAs in human plasma [109] was invalidated by a reanalysis of the dataset, which suggested that their presence was an artifact [110].

All these controversies call for different hypotheses to explain plant miRNA cross-kingdom activity. It is plausible that food-derived miRNAs can affect host genes when encapsulated in plant EVs [111]. This hypothesis is supported by the fact that in this form plant miRNAs are stable after cooking processes [112] and can be isolated from different food components [113]. In addition, in many organisms, including humans, miRNAs transported by EVs can exert their regulatory role in different cellular districts [114].

Another possibility is that food-ingested plant miRNAs could influence host genes indirectly by acting on the gut microbiota. In this regard, these molecules could be degraded by RNases and microbial nucleases present in the gastrointestinal tract, providing nucleotide fragments that may be beneficial for microbiota metabolism, or the permeability of the intestinal barrier could play a major role in their absorption efficiency [115].

Finally, it is important to take into account that technical limitations in identifying plant-derived miRNAs in mammalian body fluids and differences in experimental design can impact the analysis of results. Thus, multiple aspects should be addressed to confirm with certainty that food-ingested plant miRNAs can be delivered to host tissues and regulate gene expression.

Taken together, this evidence indicates that miRNAs are important epigenetic mediators capable of integrating genetic, nutritional, and environmental signals in the regulation of metabolic pathways. Dietary miRNAs, together with nutrients and phytochemicals enclosed in vegetables present in the MD, may contribute to modulating the expression of genes involved in lipid metabolism and energy homeostasis, suggesting a possible link between diet, epigenetic regulation, and the development of metabolic diseases such as obesity. Although additional studies are needed to better investigate the cross-kingdom anti-obesogenic role of edible plant miRNAs, they may be considered a novel strategy to counteract the increase in obesity by acting on food intake modulation.

## 5. Computational Approaches to Mediterranean Diet (MD)-Driven Regulation of Obesity

Recent advances in bioinformatics have substantially enhanced the ability to explore the molecular mechanisms underlying obesity, particularly in the context of complex dietary patterns such as the MD. The MD is not defined by a single nutrient but by a synergistic combination of foods rich in polyphenols, unsaturated fatty acids, fiber, and bioactive compounds, whose biological effects emerge from coordinated regulation across multiple molecular layers. In this scenario, bioinformatics represents a critical framework to integrate high-dimensional data and decipher how MD-related exposures modulate obesity-associated pathways.

High-throughput sequencing technologies, combined with bioinformatic pipelines, have enabled the identification of dysregulated miRNAs and their downstream target genes in obesity.

Several miRNAs implicated in lipid metabolism, adipogenesis, inflammation, and insulin signaling (e.g., miR-29, miR-122, miR-143, miR-378a) have also been shown to be responsive to dietary interventions, including the MD dietary pattern. Bioinformatic integration of miRNA–mRNA interaction networks, pathway enrichment analyses, and gene regulatory network reconstruction allow the positioning of these miRNAs within MD-sensitive molecular circuits rather than considering them as isolated biomarkers. Importantly, integrative reviews indicate that circulating miRNAs may act as molecular readouts of MD-induced metabolic and inflammatory improvements, although causality in humans remains to be established [116,117].

Tools integrating miRNA–mRNA interactions [85], metabolic pathway resources (KEGG, BRENDA), and gene network reconstruction have enabled the identification of regulatory hubs linking obesity to chronic inflammation and metabolic dysfunction, highlighting these genes as key molecular targets through which Mediterranean Diet-associated foods may exert their beneficial metabolic effects. Multi-omics bioinformatics frameworks—including epigenomics, transcriptomics, proteomics, metabolomics, and microbiome profiling—have emerged as particularly powerful tools for investigating MD-related heterogeneity in obesity [118,119].

Recent multi-omics studies have demonstrated that individual variability in response to MD interventions is strongly influenced by baseline molecular and microbiome signatures, which can be captured only through integrative computational approaches [120]. For instance, combined host genomic, metagenomic, and metabolomic analyses have been shown to explain inter-individual differences in adiposity reduction, inflammatory markers, and insulin sensitivity following long-term MD interventions [121]. These computational multi-omics strategies are highlighted in a recent study that emphasizes the importance of data integration algorithms and network inference tools in identifying clinically relevant biomarkers and regulatory modules in obesity [122].

Bioinformatics also enables the systematic investigation of diet–epigenome interactions, a key mechanism by which the MD may exert long-term metabolic benefits. Computational analyses have shown that MD components can modulate DNA methylation patterns and histone-related regulatory networks involving genes associated with lipid metabolism, inflammation, and mitochondrial function. Moreover, nutrigenomic and systems biology approaches allow the integration of dietary exposure data with epigenetic marks and miRNA expression profiles, supporting a mechanistic link between MD adherence and the attenuation of obesity-related metabolic dysfunction [118,119].

An emerging bioinformatics-driven area of interest concerns the role of diet-derived and plant microRNAs. Computational tools have been instrumental in assessing sequence conservation, characterizing miRNA seed regions, predicting cross-kingdom interactions, and mapping candidate metabolic targets. Specifically, seed sequence complementarity, which represents a primary determinant of miRNA–target recognition, has been extensively used to identify putative human transcripts susceptible to regulation by plant-derived miRNAs [123,124,125]. In this context, a bioinformatic study has demonstrated that plant miRNAs can regulate human long non-coding RNAs through sequence-specific interactions. In particular, two plant miRNAs from *Medicago truncatula* (mtr-miR-5754) and *Glycine max* (gma-miR-4995) directly target the oncogenic long non-coding RNAs *MALAT1* and *NEAT1*, thereby reducing cancer cell proliferation via a cross-kingdom regulatory mechanism [97].

The discovery that nut-derived miRNAs can target Tnfrsf1a and attenuate inflammatory signaling, thereby improving insulin sensitivity in adipocytes and obese mice, was driven by computational target prediction analyses performed through the combined use of BLAST-based sequence alignment (BLAST v2.2.30) and RNAhybrid to evaluate the thermodynamic stability of miRNA–mRNA duplexes [82]. Finally, bioinformatics supports the development of personalized therapeutic strategies, including the design of miRNA mimics, antagomiRs, and edible nanoparticle-based delivery systems. In addition, the use of machine learning (ML) models on multi-omics datasets enables the stratification of individuals based on molecular obesity signatures and improves the prediction of therapeutic responses. Bioinformatics-driven ML applications have been used to classify obesity phenotypes, identify predictive molecular features, and model metabolic outcomes using neural networks and decision tree-based algorithms, demonstrating the potential of computational approaches for precision medicine interventions in metabolic disorders. Future research directions should prioritize longitudinal MD intervention studies combined with standardized multi-omics data generation and advanced bioinformatic integration. The application of causal inference models, network perturbation analysis, and explainable ML approaches will be essential to move beyond correlation and to identify actionable regulatory nodes linking MD adherence to obesity prevention and metabolic health.

## 6. Conclusions

The Mediterranean Diet influences multiple metabolic pathways simultaneously by modulating lipid metabolism, gut microbiota composition, and redox homeostasis. Western diets, characterized by high consumption of ultra-processed foods and sugar-sweetened beverages, promote fat accumulation, increase oxidative stress and obesity-related comorbidities. The plant-based foods of the MD can prevent the increase in obesity through the action of bioactive molecules like polyphenols or through the cross-kingdom activity of plant miRNAs.

Importantly, advances in bioinformatics now allow the integration of multi-omics datasets, epigenomic, transcriptomic, proteomic, metabolomic, and microbiome layers, to unravel the molecular mechanisms through which dietary patterns such as the MD exert their protective metabolic effects. These computational approaches have been essential for uncovering microRNAs and regulatory networks that mediate obesity-related inflammation, insulin resistance, and adipocyte dysfunction, thereby providing a mechanistic bridge between nutritional patterns and metabolic phenotypes. Furthermore, bioinformatic analyses have elucidated the contribution of both endogenous and plant-derived miRNAs to metabolic homeostasis, revealing cross-kingdom interactions and nutrigenomic pathways relevant to the MD’s anti-obesogenic action. In conclusion, adopting the Mediterranean Diet represents an effective strategy to support metabolic health and prevent lipid disturbances associated with obesity, while bioinformatic multi-omics approaches offer new opportunities to mechanistically validate these benefits and develop personalized nutrition strategies grounded in molecular evidence.

## Data Availability

No new data were created or analyzed in this study. Data sharing is not applicable to this article.
